# Hybrid cluster precursors of the LaZrO insulator for transistors: lowering the processing temperature

**DOI:** 10.1038/s41598-018-24292-4

**Published:** 2018-04-12

**Authors:** Peixin Zhu, Jinwang Li, Phan Trong Tue, Satoshi Inoue, Tatsuya Shimoda

**Affiliations:** 1 Center for Single Nanoscale Innovative Devices, Japan Advanced Institute of Science and Technology (JAIST), 2-13 Asahidai, Nomi, Ishikawa, 923-1211 Japan; 2 Core Functionalities Development Center, Central Research Laboratories, DIC Corporation, 631 Sakado, Sakura, Chiba, 285-8668 Japan; 3 Japan Science and Technology Agency (JST), ERATO, Shimoda Nano-Liquid Process Project, 2-13 Asahidai, Nomi, Ishikawa, 923-1211 Japan; 4 School of Materials Science, Japan Advanced Institute of Science and Technology (JAIST), 1-1 Asahidai, Nomi, Ishikawa, 923-1292 Japan

## Abstract

Solution processing of ternary and multinary amorphous metal oxide insulators at processing temperatures below 250 °C remains challenging. Here, we report that the synthesis of a hybrid cluster structure, where the metal oxide core is coordinated by ligands and the different metal elements are incorporated into one core, is an effective strategy for the low-temperature processing of the ternary LaZrO insulator. Solvothermal treatment at 160–180 °C facilitated the development of a cluster structure. From the cluster precursor, high-performance insulating LaZrO films were obtained at 200 °C under the irradiation of ultraviolet light. The analysis data indicate that the solvothermal treatment led to structural unification of the metal oxide network and facilitated stabilization of the residual organic ingredients in UV annealing, which both contributed to the improved insulating properties of LaZrO. Together with a solution-processed channel, we have been able to fabricate LaZrO-based transistors at 200 °C. Though the channel material has not been optimized, the transistor have showed a low gate leakage current around 10 pA at an operating voltage of 15 V, an on/off ratio of near 10^6^, a field-effect saturation mobility of 0.37 cm^2^ V^−1^ s^−1^, a subthreshold swing factor of 0.61 V decade^−1^.

## Introduction

Low-temperature solution processing of oxide thin-film transistors (TFTs) has attracted significant interest because it can allow the use of low-cost, flexible, and transparent plastic substrates in conjunction with facile solution deposition or printing techniques. Hitherto, several low-temperature methodologies have been developed, namely the “sol-gel on chip” process^1^, solution combustion processing^2–4^, ultraviolet (UV) light-assisted (photochemical) annealing^5–7^, as well as the very recently reported methods employing redox reactions^8^ or aqueous solutions^9^. Using these techniques, processing at 250 °C or lower temperatures is possible and processing at as low as 150 °C through UV annealing has been reported.5–67. These advances are significant, but the processable materials, especially insulators, are limited. Most of these processes have been applied to the semiconducting channel materials in transistors, and only the UV-annealing and aqueous solution techniques have been applied to alumina, 6, 7, 9 zirconia, 7 and hafnia 7 insulators. These insulators are binary oxides, among which alumina can be doped with zirconium at an ultimate concentration of a few percent. For practical applications, it is necessary to expand the types of insulating materials processable at low temperatures. In particular, low-temperature processing routes for ternary or multinary insulating metal oxides remain unexploited and should also be developed. One practical reason is that incorporation of more elements allows tailoring the properties in a wider range and may lead to finding new functionalities. Compared to binary oxides, ternary and multinary oxides may have more stable amorphous structures because the arrangement of three or more types of atoms into crystals would be more difficult, as in our LaZrO. Amorphous structures are desirable for low leakage currents, smooth surfaces, and sharp insulator–channel interfaces, all of which lead to high transistor performances.

For ternary and multinary oxides, the preferential decomposition of one metal-precursor compound over another can result in compositional segregation, even though the compositional distribution is on the molecular scale in the precursor. This imposes additional difficulties for low-temperature processing. The length scale of compositional heterogeneity can be up to nanometers, and high temperatures are needed to decrease it^10^. Compositional heterogeneity significantly decreases the insulating properties of the material^11^.

We have investigated the solution-phase synthesis of oxide structures containing two or more metals. In our previous studies, we observed that hybrid cluster structures are typically formed in solution precursors of oxides such as indium oxide^12^ and ruthenium oxide^13^. The hybrid clusters have an inorganic core (M-O-M) chelated by organic ligands. For instance, we observed typical cores having seven indium atoms and three ruthenium atoms, respectively, in the above examples. Here, we propose synthesizing such a cluster core containing two or more metal elements with a structure similar to that of the desired final oxide, and then, using the core as a building block for film deposition. In this method, the preferential decomposition of one metal compound over the others, which causes compositional segregation, is not expected to occur because the different metal elements have already been combined into one core and thermodynamically stabilized. Hence, the decomposition and densification are similar to those occurring in a binary metal oxide system. Compared with nanoparticles, such clusters are preferred because nanoparticles are relatively large and only compact into porous structures unless sintered at high temperatures. The core of our proposed cluster typically has less than or around ten metal atoms and is expected to be less than or around one nanometer in size. The previously reported strategies can be used to remove the ligands at low temperatures.

We studied this method for the ternary insulating material LaZrO, which has a relative dielectric constant (~20 or higher, depending on processing, impurities, etc.) around three times higher than that of alumina, and has resulted in high TFT performance when used as an amorphous gate insulator in our studies^12,14,15^. In an organic acid solution, a cluster structure coordinated by carboxylate ligands with six zirconium atoms (Zr6) in the oxide core has been reported^16,17^. We further synthesized LaZrO clusters by adding La and achieved uniform and stable clusters under solvothermal conditions. The well-developed clusters obtained after solvothermal treatment can form high-quality insulating films by annealing at 200 °C, with the assistance of UV light irradiation. The structure of the clusters, as well as their uniformity and stability, not only influences the insulating properties of low-temperature deposited films but also significantly affects the structures and properties of films deposited at high temperatures, as presented in our previous paper^18^. In that paper^18^, we have reported detailed structural analyses of solutions, gels, and solids, and show how the precursor structure determines the final solid structure after annealing at high temperature. These results also indicate the importance of detailed investigation into precursor structure and its optimization, which are rarely performed. In the current paper, we focus on the processing of LaZrO films at low temperatures and demonstrate its application as a gate insulator in transistors fabricated at 200 °C.

## Experimental Methods

### Synthesis of Precursor Solutions

In a typical experiment, lanthanum(III) acetate 1.5-hydrate (0.686 g, 99.99%, Kanto Kagaku) and zirconium(IV) butoxide solution (0.959 g, 80 wt. % in 1-butanol, Aldrich) were each dissolved in appropriate amounts of propionic acid (>99.0%, Kanto Chemical) in capped glass vials with magnetic stirring for 30 min on a hotplate set at 110 °C to produce 0.2 mol/kg La and Zr solutions (10 g of each). The Zr compound was handled in a dry nitrogen-filled glovebox before being sealed in the vial. After cooling to room temperature, the two solutions were mixed to obtain LaZrO precursor solutions with La/Zr molar ratio of 3/7 or 1/1. For solvothermal treatment, 10 g of LaZrO precursor solution was sealed in an autoclave with a 50 ml PTFE inner container (HUT-50, San-Ai Kagaku Co. Ltd.) and heated at 160–180 °C on an autoclave heater (RDV-TMS-50, San-Ai Kagaku Co. Ltd.) for 2–5 h with magnetic stirring. The selection of the reagents and solvents is decided based on our previous study that this system formed La-Zr clusters that developed toward structural unification after solvothermal treatment. In our preliminary experiments, we had used nitrate reagents in 2-methoxyethanol for LaZrO and did not find such a feature, and the properties of the resulting transistors were less stable.

To prepare the solution for the semiconducting indium oxide (InO) channel material, indium(III) nitrate hydrate (0.355 g, 99.999%, Aldrich) was dissolved in deionized water (5 g, >99.0%, Kanto Chemical) in a capped glass vial with stirring at room temperature for 1 h.

### Film Deposition and Device Fabrication

All solutions used for film deposition were filtered through a 0.2-μm-pore filter. Pt(200 nm)/SiO_2_(200 nm)/Si substrates, typically 2 cm × 2 cm in size, were cleaned using oxygen plasma before deposition. The LaZrO precursor solution was spin-coated onto a substrate at 2000 rpm for 25 s, dried at 100 °C or 150 °C on a metallic hotplate for 5 min, and then annealed at a substrate temperature of 150–250 °C for a typical time of 10 min under UV irradiation (wavelengths 184.9 (~10%) nm and 253.7 nm (~90%), generated by a low-pressure mercury lamp, with an intensity of ~ 10 mW cm^−2^) in an oxygen flow (5 L/min × 2 inlets) using the UV-300H system (Samco Inc.), with O_3_ produced by an O_3_ generator in the system. The spacing between the UV lamp and the samples was around 1 cm. To study the effect of atmosphere, the UV-annealing was also performed in a nitrogen flow (5 L/min × 2 inlets) without turning on the O_3_ generator under otherwise same conditions. The spin-coating and annealing cycles were performed up to 5 times to achieve a film thickness of around 120 nm.

For comparison, samples only thermally annealed at 500 °C and 600 °C were prepared. These samples were dried at 250 °C on a metallic hotplate for 5 min after spin-coating. The spin-coating and drying procedure was performed 5 times for a final film thickness of around 120 nm. The samples were then pre-annealed at 400 °C for 5 min on a ceramic hotplate in air, followed by annealing at 500 °C or 600 °C for 20 min under an oxygen flow in a rapid thermal annealing furnace.

For the measurement of insulating properties (current–voltage and dielectric constant–frequency relations), Pt top electrodes (100 nm thick and 200–500 μm in diameter) were deposited through a metal mask using radio-frequency (RF) plasma sputtering at room temperature to form a capacitor structure. Post-annealing was performed at 200 °C for 20 min in air.

TFTs were fabricated using a conventional photolithography patterning process. First, a Pt gate-electrode pattern (100 nm thick) was prepared using a RF plasma sputtering (at room temperature) and lift-off process on a SiO_2_/Si substrate. A RF plasma-sputtered Ti adherence layer (10 nm thick) was used between the Pt and the SiO_2_. Second, the LaZrO insulator was deposited as described above. Third, the InO channel layer was deposited using spin-coating (3000 rpm, 20 s) followed by drying (100 °C, 5 min) and UV-annealing at 200 °C for 10 min under a nitrogen flow (UV-300H system, Samco Inc.) with the O_3_ generator switched off. The use of a nitrogen atmosphere instead of an ozone and oxygen atmosphere was based on our preliminary experiments on a transistor with an InO(channel)/SiO2(insulator)/p++Si(gate) structure, where nitrogen gave rise to higher properties. Fourth, Pt (100 nm thick)/ITO (5% Sn-doped indium oxide, ~60 nm) source and drain electrodes were prepared using the sputtering and lift-off process. Fifth, the InO channel was patterned by wet etching through a photolithographically deposited resist mask. The resist mask was thereafter removed via dissolving in a solvent. Finally, the TFTs were post-annealed at 200 °C for 2 h on a hotplate in air.

### Measurements

UV–visible absorption measurements for the solution samples were performed using a V-630 spectrophotometer (JASCO Corporation) with a 10 mm-path-length quartz cell. Film thicknesses were evaluated through an ellipsometry (Sopra GES-5E Ellipsometer, SEMILAB Japan K.K.). Film densities were measured using the X-ray reflectivity (XRR) method (X’Pert PRO MRD, PANalytical) for the LZO/Pt/SiO_2_/Si samples. The XRR data fitting was performed using the analysis software of the XRR system. Ideal pure oxide compositions of La_3_Zr_7_O_18.5_ and La_5_Zr_5_O_17.5_ were used in the fitting. The thickness values from ellipsometry analysis were input as initial parameters. Both density and thickness values were fitted. The resulted thickness values were similar to the ones from ellipsometry analysis. The uncertainty of the obtained density values was around ±0.05 g cm^−3^. A typical resulted plot of the fitting is presented in Figure S1. X-ray photoelectron spectroscopy (XPS) analysis was performed using a Kratos AXIS-ULTRA DLD system (Shimadzu). The surface morphologies were investigated through an atomic force microscopy (AFM; NanoNavi, SII). Elemental compositions of the thin films were obtained from combinational Rutherford backscattering spectrometry, nuclear reaction analysis, and hydrogen forward scattering spectrometry on Pelletron 3SDH (National Electrostatics Corp.) at Toray Research Center, Inc., Japan, with estimated precisions of ±0.5 atom% for C and H, ±1 atom% for La and Zr, and ±4 atom% for O. Impedance measurements for Pt/LaZrO/Pt capacitor samples were performed using SI 1260 Impedance/Gain-Phase Analyzer (Solartron Analytical). The current–voltage characteristics of these capacitor samples and the TFT performance were measured using a 4155C Semiconductor Parameter Analyzer (Agilent).

## Results and Discussion

### Formation of Hybrid Clusters

The details have been presented in our previous paper^18^. A simple description is given here for the convenience of readers. We dissolved lanthanum acetate and zirconium butoxide in propionic acid below 110 °C. In the absence of La, this system forms the Zr6 cluster^16^. We used La/Zr atomic ratios of 1/1 (LZ55) and 3/7 (LZ37) based on our observation that Zr-rich compositions led to higher TFT performance. A simple and convincing evidence for the cluster formation is that the thermal decomposition temperature of the LaZrO precursor (two main exothermic peaks at 327 °C and 345 °C) is lower than those of both Zr-only (350 °C) and La-only (365 °C) precursors, indicating that the Zr and La components were not simply mechanically mixed.

However, these LaZrO precursors were found to result in films with poor insulating properties when annealed at both low (see below) and high (see ref.^18^) temperatures. This is attributed to a low degree of cluster development under the above synthesis conditions. Therefore, we further heated the solutions under solvothermal conditions in an autoclave (AC) at temperatures (160–180 °C) higher than the boiling temperature of the solvent (propionic acid, b.p. 141.2 °C) in order to facilitate the development of the clusters. This led to clusters of improved uniformity and stability, as indicated by thermal analysis^18^. The higher uniformity was also supported by synchrotron X-ray diffraction measurements, which showed that solvothermal treatment of the precursor solution led to the disappearance of a second phase that was otherwise present in the samples annealed at 500 °C.

### Light Absorption Analysis of Solutions

**Figure 1:**
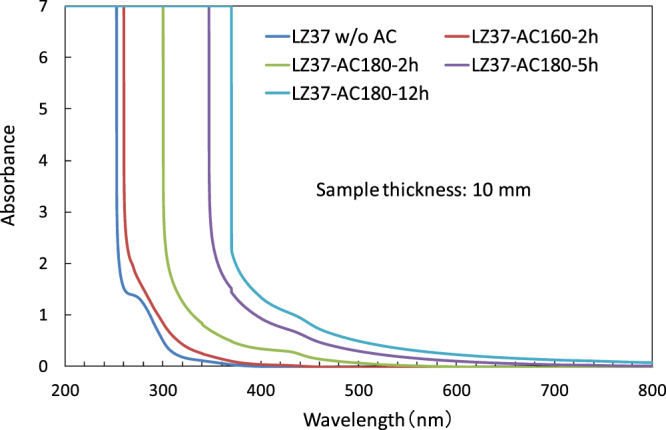
UV–visible spectra of LZ37 solutions.

As a result of solvothermal treatment, the appearance of the precursor solution changed from colorless to slightly yellowish, indicating that the light absorption range widened into the visible region. The color became deeper with increasing duration and temperatures of the solvothermal treatment. The UV–visible light absorption of the solutions was measured and the results (Fig. 1 and Figure S2) showed that the absorption range was significantly widened to include longer wavelengths in the visible region after the solvothermal treatment. The absorption reached the measurement limit of the detector at 252 nm before solvothermal treatment (without AC or w/o AC), while the measurement limit was reached at 260 nm, 300 nm, 348 nm, and 370 nm after the treatment at 160 °C for 2 h (AC160-2h), 180 °C for 2 h (AC180-2h), 180 °C for 5 h (AC180-5h), and 180 °C for 12 h (AC180-12h), respectively. Such changes in absorption were considered to be a result of the structural reorganization of the cluster cores. Hence, the solvothermally treated solutions were found to have a highly enhanced UV absorption ability, which may facilitate the decomposition of their organic ligands under UV irradiation.

### Low-Temperature UV-Annealing of Films and Their Characterization

The UV light we applied is in the deep UV range that has energies (647 and 472 in kJ mol^−1^ for UV light at wavelengths of 184.9 nm and 253.7 nm, respectively) high enough to decompose typical bonds of impurities in the precursors (e.g., energies in kJ mol^−1^ for C-C is 347.7, C-O 351.5, O-H 462.8). Thus, deep UV-annealing (without ozone) has been applied for the low-temperature solution deposition of films of aluminum oxide and zirconium oxide from nitrate or organic precursors.5-,67 On the other hand, in the presence of ozone and O_2_, active atomic oxygen (O), with strong oxidation ability, is produced according to the Chapman mechanism (O_2_ + UV light < 240 nm → O + O and O_2_ + O → O_3_; O_3_ + UV light < 320 nm → O_2_ + O)^19^. It can oxidatively decompose impurities in the precursors. Therefore, one may expect that the use of ozone and O_2_ (UV/O_3_ annealing) would facilitate solidification. However, it was also reported that UV annealing without ozone and O_2_ was more effective,5-67 possibly because that the absorption of UV light by ozone and O_2_ reduced the intensity of UV rays reaching into the films. In our study, we found UV/O_3_ annealing resulted in higher electrical properties. In the following, we will start from UV/O_3_ annealing and will compare the effects of annealing atmospheres as well as other parameters (with and without solvothermal treatment, La/Zr ratio, and thermal *versus* UV annealing).

#### Low-temperature deposition using UV/O_3_ annealing

**Figure 2:**
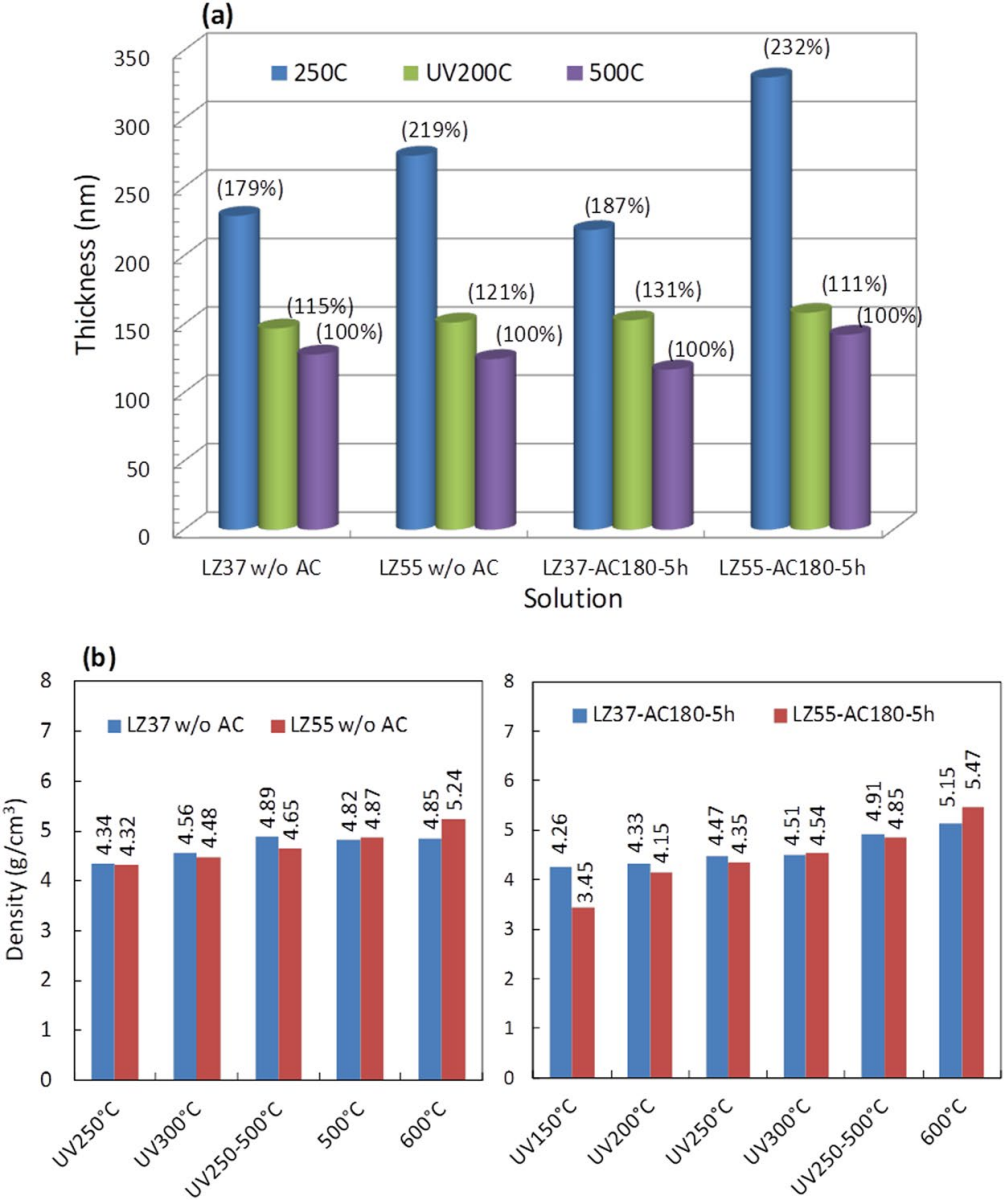
(**a**) Thickness of LaZrO films UV/O_3_-annealed at 200 °C and thermally annealed at 250 °C and 500 °C. The numbers in parentheses are the relative thickness values normalized by the thickness of the film thermally annealed at 500 °C. (**b**) Densities of LaZrO films. UV250–500 °C indicates UV/O_3_ annealing at 250 °C followed by thermal annealing at 500 °C. Left: Without solvothermal treatment of the solution; right: with solvothermal treatment. For UV-annealed samples, the drying temperature in film deposition was 100 °C.

UV/O_3_ annealing of films spin-coated from precursor solutions with and without solvothermal treatment was performed. Fig. 2(a) presents the thicknesses of the resulting films, together with those of reference samples that were only thermally annealed (without applying UV light) at 250 °C and 500 °C. The thickness decreased as a result of the decomposition of the organic components. The sample thermally annealed at 500 °C was the thinnest for each solution, indicating the most complete decomposition and densification. Such an annealing temperature is commonly applied for TFT fabrication. Compared with this sample, the sample thermally annealed at 250 °C was around 1.8–2.3 times thick, indicating that a substantial amount of organic material remained. In fact, thermal decomposition required temperatures higher than 300 °C according to the thermal analysis^18^. When UV light was applied, the films annealed at a low temperature of 200 °C were only 11–31% thicker than those thermally annealed at 500 °C. Therefore, under UV light, it is promising to fabricate LZO insulator at just 200 °C.

The densities of the films are presented in Fig. 2(b). The solvothermally treated samples had slightly higher (2.9% in average) film densities than the films without UV treatment with one exception (LZ37-UV300 °C), namely enhancement of density was observed in seven samples among eight ones. And the solvothermally treated LZ37 films are slightly denser than the corresponding LZ55 films under the conditions of UV200 °C, UV300 °C and UV250–500 °C whereas the latter is denser than the former after the annealing at 600 °C.

The LZ37 films UV-annealed at 200–300 °C had densities in the range 4.3–4.5 g cm^−3^, which corresponds to only 70–74% of the density of LaZrO crystals (assumed to be 6.1 g cm^−3^ based on the values of La_2_Zr_2_O_7_, 6.06 g cm^−3^, and La_0.1_Zr_0.9_O_1.95_, 6.1–6.3 g cm^−3^)^20,21^. Even so, they may be applicable in TFTs, considering that even films annealed at 500–600 °C were only 80–84% dense (4.9–5.2 g cm^−3^).

#### Dielectric properties and the influencing parameters

**Figure 3:**
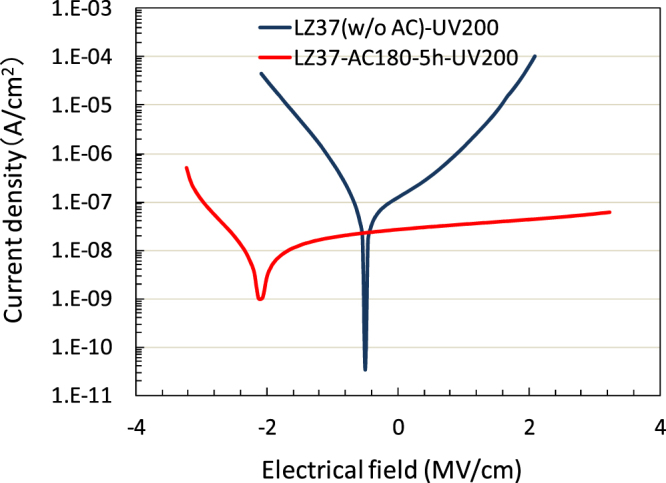
Dependence of current density on electrical field for the LZ37 films UV/O_3_-annealed at 200 °C from solutions with and without solvothermal treatment. The drying temperature in film deposition was 100 °C.

We then evaluated the insulating property of the LZ37 films UV-annealed at 200 °C, prepared from solutions with or without solvothermal treatment. The dependence of leakage current density against the applied electrical field is shown in Fig. 3. An excellent insulating ability (~10^−8^ A cm^−2^ at 2 MV cm^−1^) of the film from the solvothermally treated solution was achieved. Without solvothermal treatment of a solution, the current density is several orders higher, indicating significant effect of solvothermal treatment on the improvement of the film properties, as also observed in the high-temperature annealed films^18^.

Because of higher properties of films from solvothermally treated solutions, the following experiments were designed for more detailed study of films from such solutions, if not otherwise indicated (the solutions used are indicated in the figure captions).

**Figure 4:**
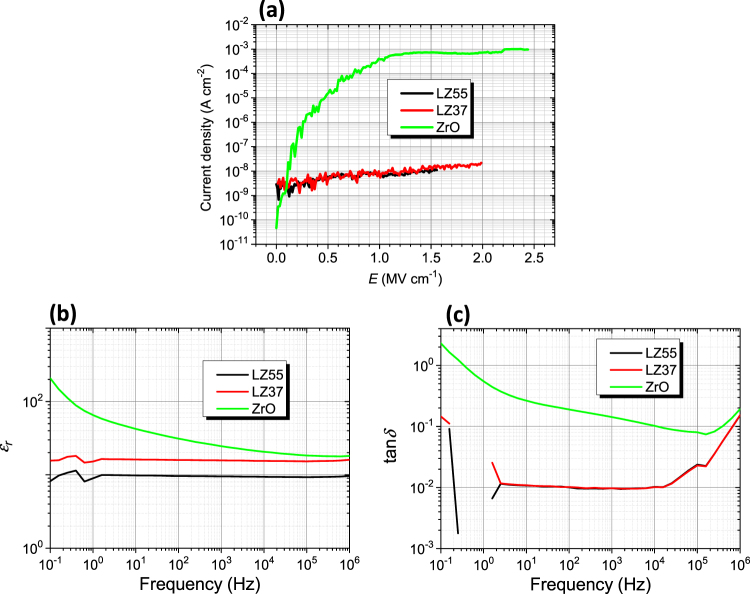
Dielectric properties of films UV/O_3_-annealed at 200 °C, showing the effect of La/Zr ratio. (**a**) Leakage current density against electric field. (**b**) Dielectric constant and (**c**) dielectric loss against frequency. The films were prepared from solutions LZ37-AC180-5h, LZ55-AC180-5h, and ZrO solution without solvothermal treatment, respectively. Solvothermal treatment of the ZrO solution led to precipitation. The drying temperature in film deposition was 100 °C.

The effects of La/Zr ratio on the dielectric properties are presented in Fig. 4, where the three films have La/Zr ratios of 5/5 (LZ55), 3/7 (LZ37), and 0/10 (ZrO), respectively. We also prepared a film with La/Zr = 10/0 (LaO), but the material failed to form smooth films (Figure S3), which is possibly related to the hygroscopic property of lanthanum oxide that may lead to unstable and easily peeling films. From Fig. 4, one finds that the ZrO film had poor dielectric properties (several orders higher in leakage current density than LZ37 and LZ55, high dependence of dielectric constant on frequency, and large tan*δ* values). This is because that un-doped ZrO_2_ crystallizes even at near room temperature^22,23^, i.e., its amorphous phase is unstable, leading to heterogeneity of the structure and formation of leaking path. Concerning LZ55, the as-prepared film was smooth and showed high dielectric properties, but during later TFT fabrication, it was peeling off from a substrate, which can be attributed to its excessive content of La. As reported in our previous paper^18^, the structure of our LaZrO is amorphous in long range but have cubic ZrO_2_ structure in short-range (1–2 nm). Such cubic structure is stabilized by La as a dopant. For LZ37, almost all La atoms have entered the structure as an alloying element when the solution was solvothermally treated, while for LZ55, partial La atoms remained outside the structure and form a second phase. Such a second La-rich phase may be unstable (like LaO) and lead to peeling off of the film. According to these results, a La/Zr ratio of 3/7 is the optimum metal composition in our samples.

**Figure 5:**
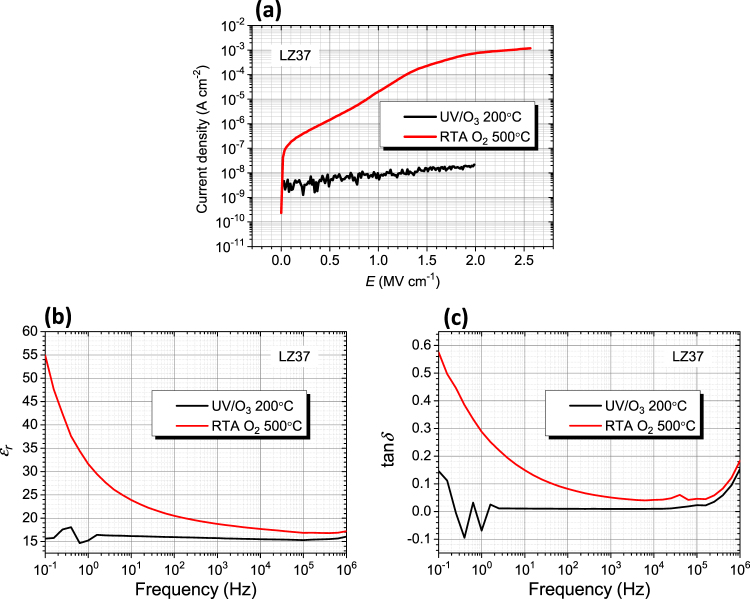
Dielectric properties of LZ37 films, one UV/O_3_-annealed at 200 °C and the other thermally annealed at 500 °C. (**a**) Leakage current density against electric field. (**b**) Dielectric constant and (**c**) dielectric loss against frequency. The films were prepared from the solution LZ37-AC180-5h. The drying temperature in film deposition was 100 °C.

In comparison of the low-temperature (200 °C) UV-annealed film and the high-temperature (500 °C) thermally annealed film (Fig. 5), we find that the former have even better dielectric properties. This can be caused by two structural differences. One is structural evolution to ZrO_2_ crystals at the elevated temperature of 500 °C, leading to phase separation. The other is destruction of the O-C bonding at 500 °C. The O-C bonding is considered to stabilize the residual carbon atoms (see the XPS analysis below for details).

**Figure 6:**
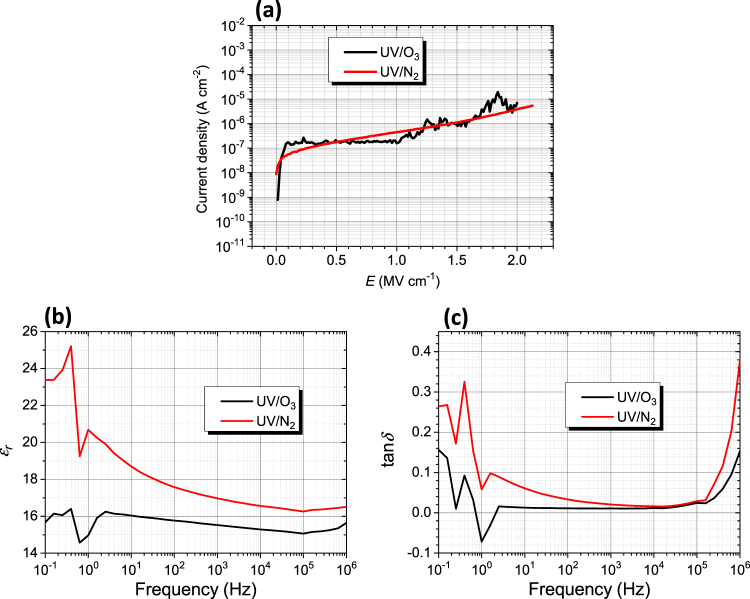
Dielectric properties of LZ37 films, one UV/O_3_-annealed and the other UV/N_2_-annealed, at the same temperature of 200 °C. (**a**) Leakage current density against electric field. (**b**) Dielectric constant and (**c**) dielectric loss against frequency. The films were prepared from the solution LZ37-AC180-5h. The drying temperature in film deposition was 150 °C.

We also studied the effect of annealing atmosphere. Fig. 6 shows the compared properties of two films, one was UV/O_3_-annealed and the other is UV/N_2_-annealed, under the same temperature and time conditions. The UV/O_3_-annealed film displays superior dielectric properties (less frequency dependence of dielectric constant and small tan*δ* values). Therefore, for our LaZrO precursors, UV/O_3_ annealing is preferred to UV/N_2_ annealing. One possible reason is that an oxygen environment facilitates oxidation of the organics while providing sufficient oxygen in the structure, leading to less oxygen vacancies.

Note that the drying temperatures in spin-coating deposition for samples in Figs 5 and 6 are different: 100 °C in Fig. 5 and 150 °Cs in Fig. 6, as indicated in the figure captions. Here, we find that the drying temperature has an effect on the insulating properties. Comparing the two LZ37 samples in these two figures, the drying at 100 °C led to higher insulating properties. However, as will be presented below, for application in TFT, drying at 150 °C gave better TFT performances. We do not know the mechanism of this effect of drying temperature, which deserves study.

#### XPS analysis

**Figure 7:**
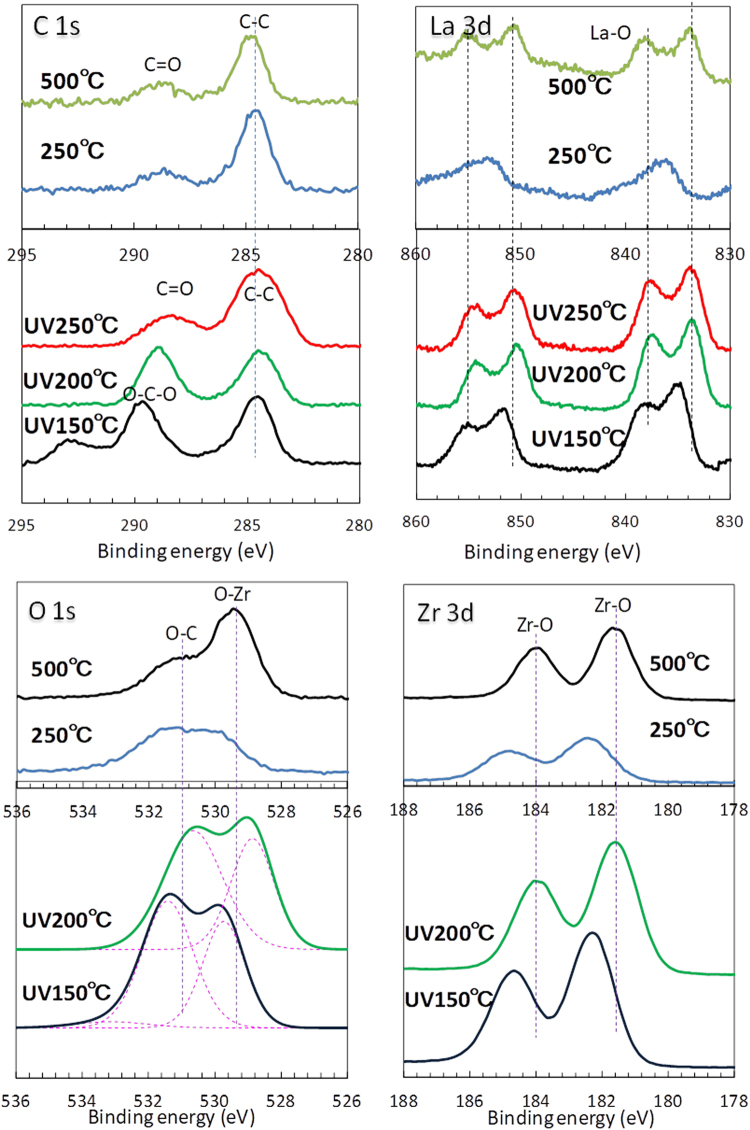
XPS analysis of the LaZrO films obtained from the solvothermally treated solution LZ37-AC180-5h. The drying temperature in film deposition was 100 °C. UV: UV/O_3_ annealing.

XPS analysis of the LZ37 films, of which solutions were solvothermally treated at 180 °C for 5 hours, was conducted to confirm the chemical states of their constituent elements (Fig. 7). Two samples were simply annealed at 250 °C and 500 °C, while the others were UV-annealed between 150 and 250 °C.

As described above, the thermal decomposition temperature of a LaZrO precursor is observed at the temperatures of 327 °C and 345 °C, where exothermic peaks were observed in the differential thermal analysis. The annealing temperature of 250 °C is lower than the decomposition temperature and that of 500 °C is sufficiently higher than it. Therefore, the XPS spectra of the LZ37 film annealed at 250 °C show the chemical states of the gel films composed of hybrid clusters whereas those of the film annealed at 500 °C show the chemical states of the LaZrO solid after decomposition.

It is amazing to see that the C 1s, La 3d, O 1s, and Zr 3d bands in the XPS spectra of LZ37 films prepared by UV annealing at 200 °C were almost similar to those of the sample thermally annealed at 500 °C. But one can find a difference between them in the O-C bonding in O 1s and C 1s. The UV-annealed films have the very strong O-C peaks compared with the 500 °C annealed film. This means that the C atoms are well terminated by oxygen and, therefore, are stabilized. That should contribute to a good dielectric property of the UV-annealed films at 200 °C shown in Fig. 5. In the case of the sample UV-annealed at 150 °C, however, it displayed bands in between those of the samples thermally annealed at 250 °C and 500 °C.

The XPS spectra of the LZ37 sample from solutions without solvothermal treatment are shown in Figure S4. These spectra show almost similar results as those solvothermally treated. The only difference was found again in the spectra of the C1s bonds of UV150 °C and UV200 °C, where much stronger oxygen-bonded C1s bands were observed for the samples prepared with the solvothermally treated solutions than those without the treatment.

From the above analyses, we observed that the UV annealing facilitated formation of C-O bonding and stabilized C atoms. These effects were further enhanced by the solvothermal treatment. Such effects must lead to the enhancement of insulating properties of LZO film.

Previously, we showed that solvothermal treatment of solutions led to compositional and structural unification of the cluster cores and this unification was maintained even after annealing at a high temperature of 500 °C^18^. The uniform amorphous structure without a second minor phase (such as observed in solids from solutions without solvothermal treatment) is advantageous for preventing formation of leakage path. In addition to the improvement of the cluster core, the solvothermal treatment of solutions also improves the orgonic ingredient to be stabilized by the UV annealing, which facilitates formation of C-O bonding. Improvements of both the cluster core and the orgonic ingredient greatly contribute to the enhanced dielectric properties of LaZrO films.

#### Surface morphology

**Figure 8:**
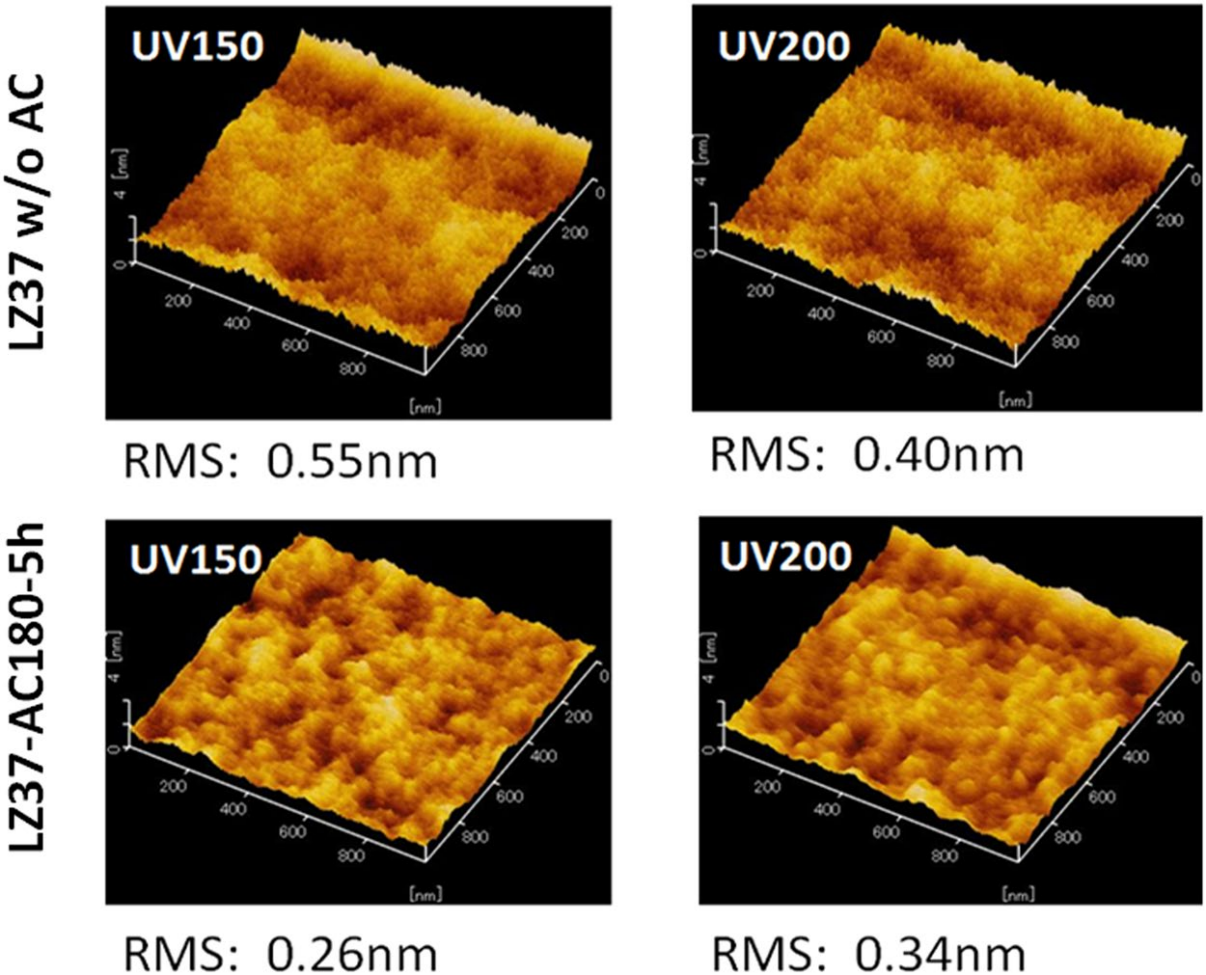
AFM images of films UV-annealed at 150 °C (UV150) and 200 °C (UV200). The drying temperature in film deposition was 100 °C.

The surface morphology of the UV-annealed films was investigated using AFM, and the resulting images are shown in Fig. 8. The average roughness, given by the root-mean-square (RMS) roughness values, was less than 0.6 nm, indicating that the surfaces were smooth. This is consistent with an amorphous film structure. In addition, the films prepared from the solvothermally treated precursor solution showed approximately 10 times larger intervals between surface peaks or between valleys, which should lead to smaller height gradients of the peaks/valleys than those in films prepared from precursor solutions without solvothermal treatment.

#### Elemental composition

Elemental analysis of the LZ37 film UV/O_3_-annealed at 200 °C from solution LZ37-180-5h gave a composition of La_0.38_ZrO_4.18_C_0.58_H_0.6_, indicating only slightly higher O and C contents than the sample thermally annealed at 400 °C (La_0.40_ZrO_3.26_C_0.24_H_0.6_). By considering the normal valence states (La 3+, Zr 4+, O 2−) of the compositional elements, the C should be bonded to O and not be in a free state. This is consistent with the above XPS analysis, where much stronger oxygen-bonded C 1s bands were observed for the sample prepared with the solvothermally treated precursor solution than without this treatment. Therefore, a uniform amorphous network structure without free carbon was considered to be formed in this sample, which also contributed to the high insulating properties.

### Low-Temperature Fabrication of Transistors

**Figure 9:**
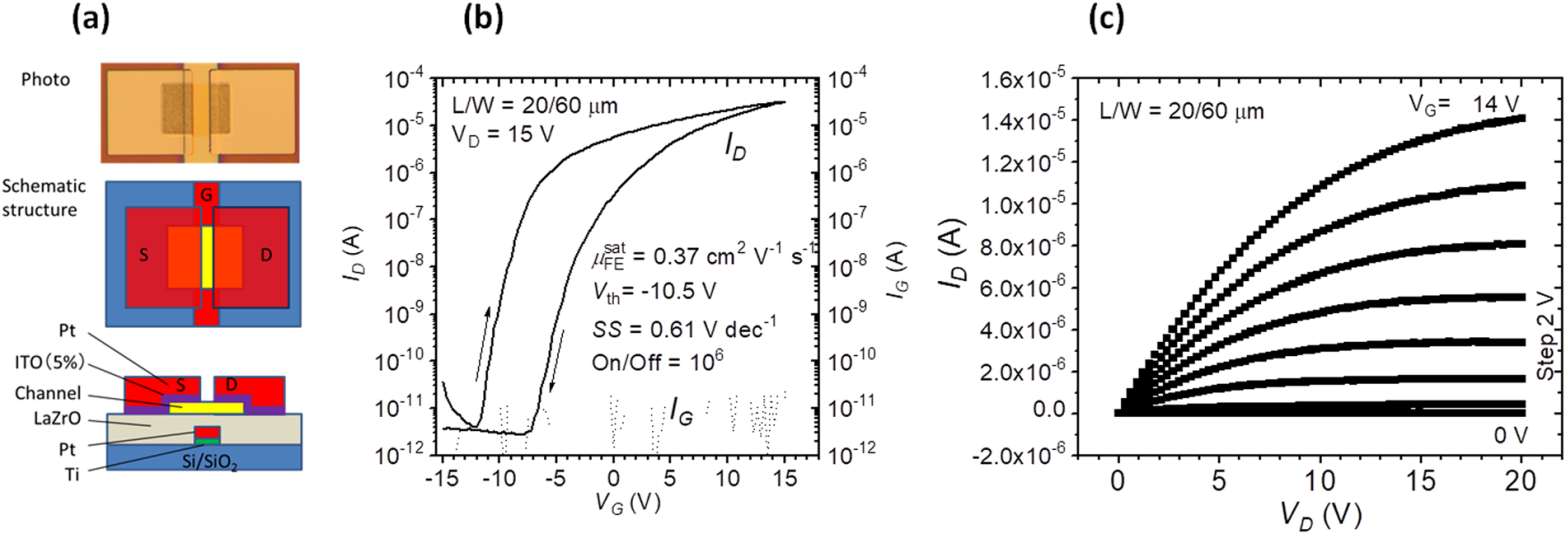
TFT prepared at 200 °C. (**a**) Structure, (**b**) transfer, and (**c**) output characteristics. The gate insulator LZ37 film was prepared from the solution LZ37-AC180-5h. In the spin-coating deposition of the LZ37 film, the drying temperature was 150 °C and the UV/O_3_-annealing was applied.

Finally, using the LZ37 film UV/O_3_-annealed at 200 °C from solution LZ37-180-5h, we fabricated a TFT. The structure is shown in Fig. 9(a). The sputtered Pt was employed as a gate electrode (G), and sputtered Pt/ITO was used as source and drain electrodes (S&D). An In_2_O_3_ (InO) film, which was prepared from an aqueous solution and UV-annealed at 200 °C, was applied as a channel. The details of the preparation procedure are described in the Experimental Methods section. After patterning by photolithography, the TFT was post-annealed at 200 °C. The performances of the TFTs are displayed in Fig. 9 (b,c) and Figure S5. Though we have observed that drying at 100 °C in LZ37 deposition resulted in higher insulating properties than at 150 °C (Figs 5 and 6), at this stage of study, we note that a drying temperature of 100 °C led to poor TFT transfer characteristics (Figure S5), while a drying temperature of 150 °C resulted in improved TFT properties (Fig. 9 (b,c)). The dielectric properties of LZ37 with drying temperatures of 100 °C, 150 °C, and 200 °C are presented in Figure S6. Drying at 200 °C resulted in poor dielectric properties (high leakage current density and high dependence of dielectric constant / tan*δ* on frequency) and drying at 100 °C led to the lowest leakage current. For TFT properties, drying at 150 °C was the best among the three temperatures. The mechanism deserves further investigation. The TFT in Fig. 9 (drying temperature of LZ37 being 150 °C) exhibited a low gate leakage current of less than 10 pA at an operating voltage of 15 V, a large on/off current ratio of near 10^6^, a field-effect saturation mobility of 0.37 cm^2^ V^−1^ s^−1^, and a subthreshold swing factor (*SS*) of 0.61 V decade^−1^. The off current of the drain (3–30 pA) and the gate leakage (~10 pA) were extremely low and comparable to those of TFTs with thermally grown SiO_2_ as the gate insulator,5 indicating excellent insulating property of the low-temperature-processed LaZrO. The *SS* value is similar to those of high-temperature-processed In-Zn-O/LaZrO (ref.^14^) and In-Zn-O/SiO_2_ (channel/gate insulator) TFTs^24^, suggesting the similar channel/gate insulator interface properties. The field-effect mobility was not satisfactorily high and the hysteresis is wide, which may be because that the channel material was not optimized. There is still much room for improving the performances after optimization.

For comparison, another TFT was also prepared using the LZ37 film UV/N_2_-annealed at 200 °C under otherwise same processing conditions. This TFT showed similar transfer characteristics with the above one concerning mobility, *SS* value, On/Off ratio, leakage current, off current, and hysteresis, upon the first measurement. However, after several times of measurement, its off current increased while the above one with the UV/O_3_-annealed LZ37 remained stable (Figure S7). This indicates that the UV/N_2_-annealed film has unstable insulating properties, consistent with the dielectric data.

## Conclusions

A methodology based on hybrid cluster precursors was proposed for low-temperature solution processing of ternary and multinary amorphous metal oxides. Hybrid clusters with metal oxide cores containing La and Zr atoms coordinated by carboxylate ligands were developed into stable and uniform structures under solvothermal conditions. These well-developed clusters showed significantly enhanced and red-shifted light absorption spectra. High-quality LaZrO insulating films with a La/Zr molar ratio of 3/7 were achieved using UV light-assisted annealing at 200 °C. The solvothermally treated solutions led to films with improved insulating and dielectric properties. This is attributed to two effects: one is the formation of a uniform and stable amorphous network structure through the improvement of cluster core and the other is enhancement of the UV annealing effect that stabilizes residual organic ingredient by facilitating formation of C-O bonding. Finally, well-performing transistors with low leakage currents were fabricated at a maximum temperature of 200 °C, together with a solution-processed channel material. After optimization of the channel material, fabrication of higher-performance oxide transistors at 200 °C or even lower temperatures would be possible. This low-temperature strategy is expected to be extended to other metal oxides, especially to ternary and multinary oxides.

## Electronic supplementary material

Supplementary information

## References

[1] Banger K (2011). Low-Temperature, High-performance solution-processed metal oxide thin-film transistors formed by a ‘sol-gel on chip’ process. Nat. Mater..

[2] Kim M, Kanatzidis M, Facchetti A, Marks T (2011). Low-temperature fabrication of high-performance metal oxide thin-film electronics via combustion processing. Nat. Mater..

[3] Hennek J, Kim M, Kanatzidis M, Facchetti A, Marks T (2012). Exploratory combustion synthesis: amorphous indium yttrium oxide for thin-film transistors. J. Am. Chem. Soc..

[4] Bae E, Kang Y, Han M, Lee C, Cho S (2014). Soluble oxide gate dielectrics prepared using the self-combustion reaction for high-performance thin-film transistors. J. Mater. Chem. C.

[5] Kim Y (2012). Flexible metal-oxide devices made by room-temperature photochemical activation of sol-gel films. Nature.

[6] Jo J (2015). Highly stable and imperceptible electronics utilizing photoactivated heterogeneous sol-gel metal-oxide dielectrics and semiconductors. Adv. Mater..

[7] Park S (2015). In-depth studies on rapid photochemical activation of various sol-gel metal oxide films for flexible transparent electronics. Adv. Func. Mater..

[8] Chen H, Rim Y, Jiang C, Yang Y (2015). Low-impurity high-performance solution-processed metal oxide semiconductors via a facile redox reaction. Chem. Mater..

[9] Rim Y, Chen H, Song T, Bae S, Yang Y (2015). Hexaaqua metal complexes for low-temperature formation of fully metal oxide thin-film transistors. Chem. Mater..

[10] Lakeman CDE, Xu ZK, Payne DA (1995). On the evolution of structure and composition in sol-gel-derived lead zirconate titanate thin layers. J. Mater. Res..

[11] Lunkenheimer P (2002). Origin of apparent colossal dielectric constants. Phys. Rev. B.

[12] Kaneda T (2014). Rheology printing for metal-oxide patterns and devices. J. Mater. Chem. C.

[13] Murakami Y, Li J, Hirose D, Kohara S, Shimoda T (2015). Solution processing of highly conductive ruthenium and ruthenium oxide thin films from ruthenium-amine complexes. J. Mater. Chem. C.

[14] Tue P (2013). High-performance solution-processed ZrInZnO thin-film transistors. IEEE Trans. Electron Devices.

[15] Tue P, Li J, Miyasako T, Inoue S, Shimoda T (2013). Low-temperature all-solution-derived amorphous oxide thin-film transistors. IEEE Electron Device Lett..

[16] Puchberger, M. *et al*. Can the clusters Zr_6_O_4_(OH)_4_(OOCR)_12_ and [Zr_6_O_4_(OH)_4_(OOCR)_12_]_2_ be converted into each other? *Europ. J. Inorg. Chem*., 3283-3293 (2006).

[17] Mos R (2012). Synthesis, crystal structure and thermal decomposition of Zr_6_O_4_(OH)_4_(CH_3_CH_2_COO)_12_. J. Analytical Appl. Pyrolysis.

[18] Li J (2016). Hybrid cluster precursors of the LaZrO insulator for transistors: properties of high-temperature-processed films and structures of solutions, gels, and solids. Sci. Rep..

[19] Jacob, D. J. *Introduction to Atmospheric Chemistry*, 162–169 (Princeton University Press, 1999).

[20] Deiseroth H-J, Müller-Buschbaum HK (1970). Ein Beitrag zur Pyrochlorstruktur an La_2_Zr_2_O_7_. Z. Anorg. Allg. Chem..

[21] Loogn C, Richardson J, Ozawa M, Kimura M (1994). Crystal structure and short-range oxygen defects in La-modified and Nd-modified ZrO_2_. J. Alloys Compounds.

[22] Park YM, Daniel J, Heeney M, Salleo A (2011). Room-temperature fabrication of ultrathin oxide gate dielectrics for low-voltage operation of organic field-effect transistors. Adv. Mater..

[23] Park YM, Desai A, Salleo A (2013). Solution-processable zirconium oxide gate dielectrics for flexible organic field effect transistors operated at low voltages. Chem. Mater..

[24] Park W-T (2015). Facile routes to improve performance of solution-processed amorphous metal oxide thin film transistors by water vapor annealing. ACS Appl. Mater. Interfaces.

