# Supplementary material for: Hybrid cluster precursors of the LaZrO insulator for transistors: lowering the processing temperature
Source: Sci Rep. 2018 Apr 12;8:5934. doi: 10.1038/s41598-018-24292-4 (PMC5897419; doi:10.1038/s41598-018-24292-4)
Supplement: Supplementary file 1 — Supplementary information [file 41598_2018_24292_MOESM1_ESM.pdf]

Hybrid cluster precursors of the LaZrO insulator for transistors: lowering the processing temperature

Peixin Zhu,[1,2] Jinwang Li,[1,3]* Phan Trong Tue,[1,3,4] Satoshi Inoue,[1,3,4] Tatsuya Shimoda[1,3,4]

[1] Center for Single Nanoscale Innovative Devices, Japan Advanced Institute of Science and Technology (JAIST), 2-13 Asahidai, Nomi, Ishikawa 923-1211, Japan

[2] Core Functionalities Development Center, Central Research Laboratories, DIC Corporation, 631 Sakado, Sakura, Chiba 285-8668, Japan

[3] Japan Science and Technology Agency (JST), ERATO, Shimoda Nano-Liquid Process Project, 2-13 Asahidai, Nomi, Ishikawa 923-1211, Japan

[4] School of Materials Science, Japan Advanced Institute of Science and Technology (JAIST), 1-1 Asahidai, Nomi, Ishikawa 923-1292, Japan

* Corresponding author. Email: jinwangli@yahoo.com

Supplementary Information

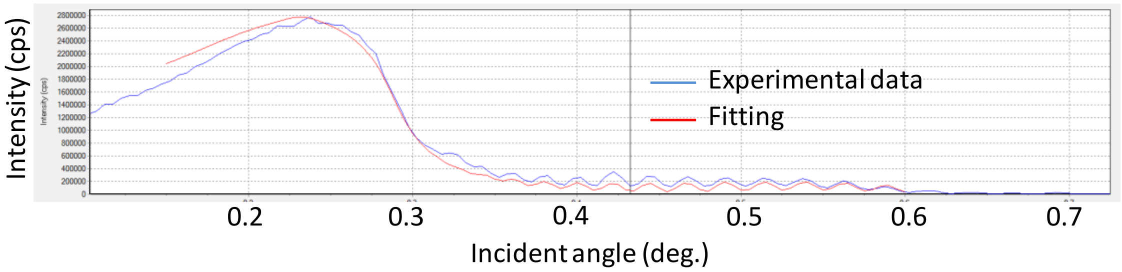

Figure S1. An example of XRR data fitting.

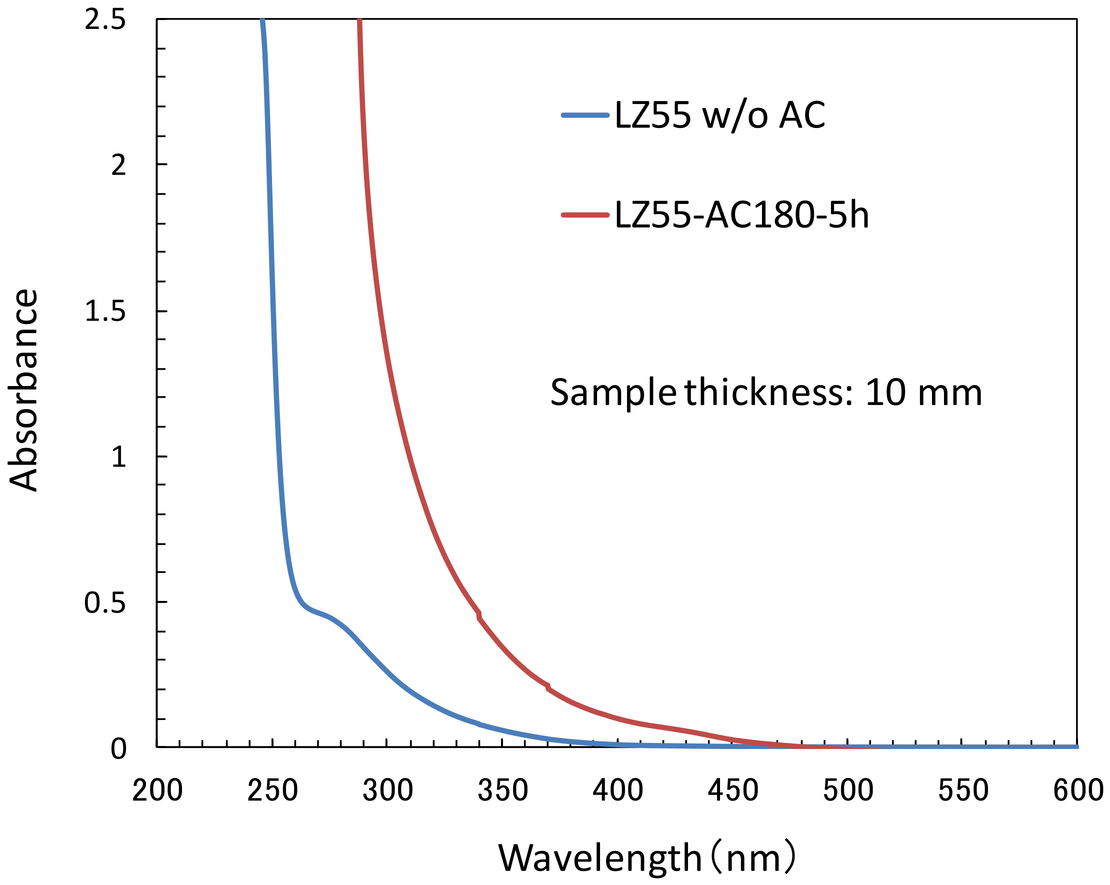

Figure S2. UV–visible spectra of LZ55 solutions.

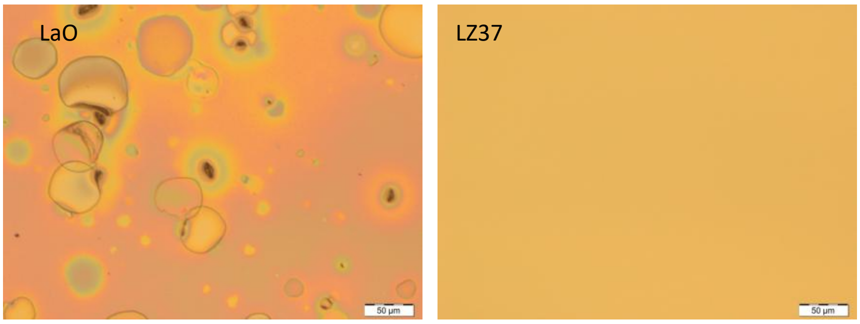

Figure S3. Photographs of LaO and LZ37 film surfaces. The films were prepared from solvothermally treated solutions LaO-AC180-5h and LZ37-AC180-5h, respectively. The drying temperature in film deposition was 100 °C.

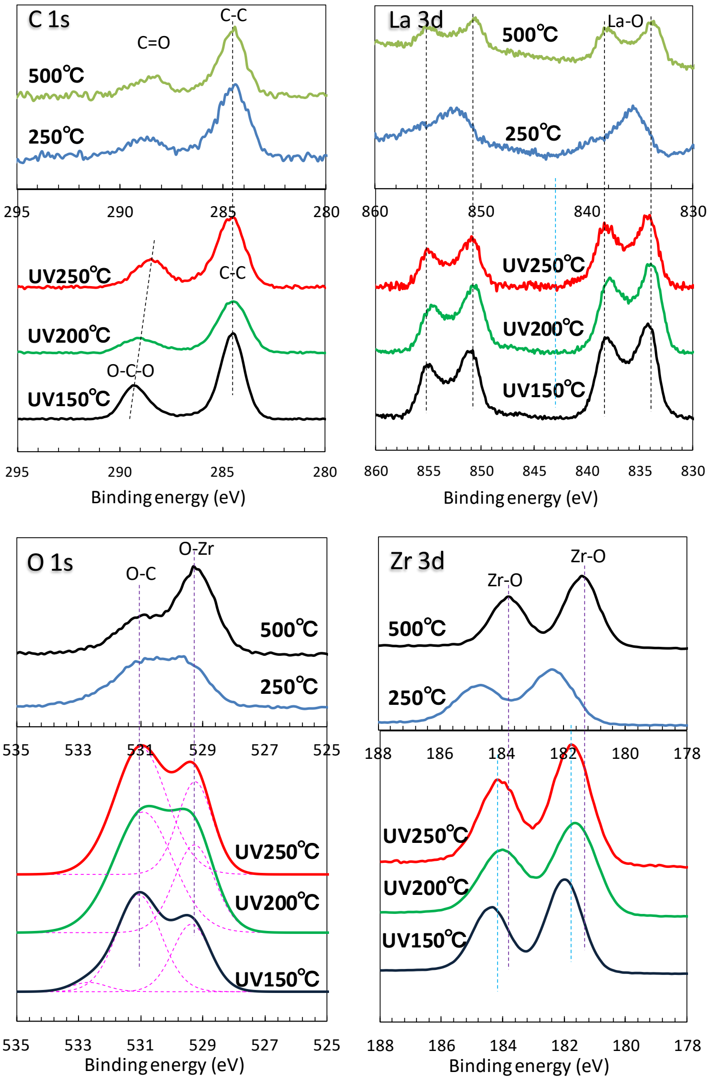

Figure S4. XPS analysis of the LaZrO films obtained without solvothermal treatment of the precursor solution. The drying temperature in film deposition was 100 °C.

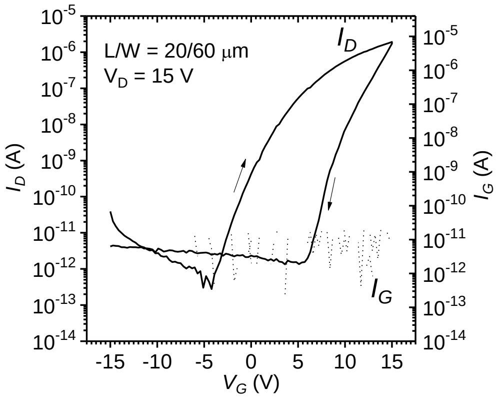

Figure S5. Transfer characteristics of a transistor with the LZ37 insulator being UV/O_3-annealed at 200 °C. The LZ37 film was prepared from the solution LZ37-AC180-5h. The drying temperature was 100°C in the spin-coating deposition of LZ37.

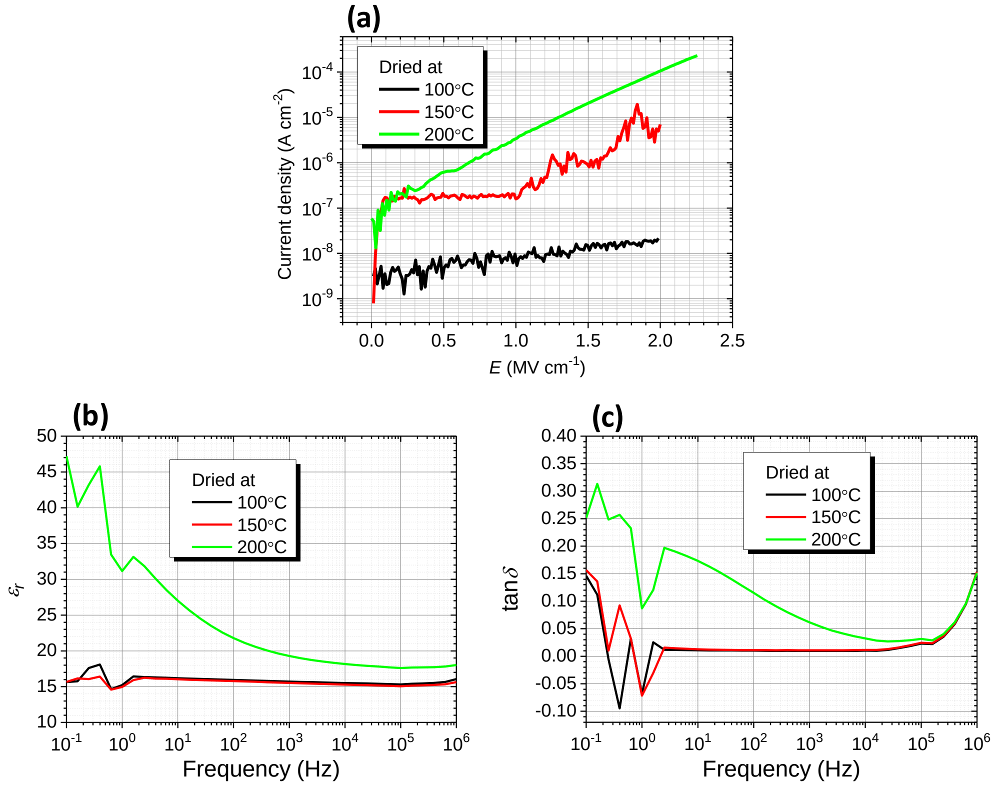

Figure S6. Dielectric properties of LZ37 films UV/O_3-annealed at 200 °C, showing the effect of drying temperature in spin-coating deposition. (a) Leakage current density against electric field. (b) Dielectric constant and (c) dielectric loss against frequency. The films were prepared from the solution LZ37-AC180-5h.

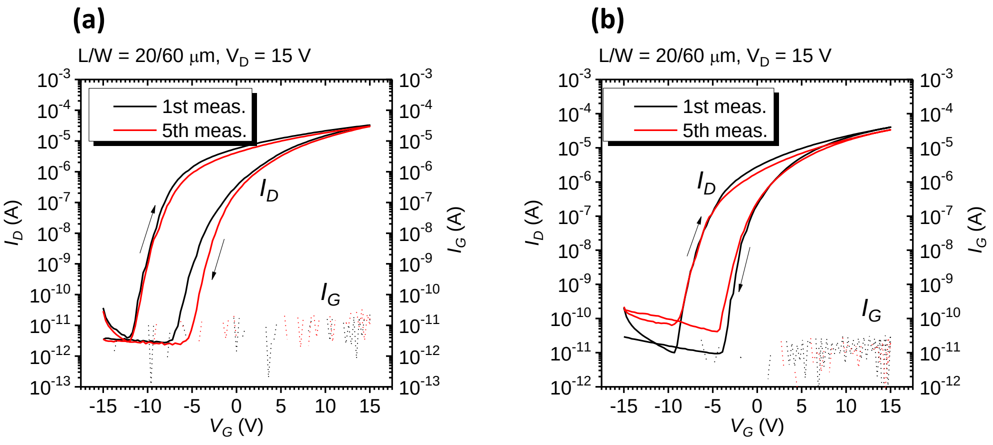

Figure S7. Comparison of the transfer characteristics of transistors with the LZ37 insulator being (a) UV/O_3-annealed and (b) UV/N_2-annealed, respectively, at 200 °C. The LZ37 films were prepared from the solution LZ37-AC180-5h. The drying temperature was 150°C in the spin-coating deposition of LZ37.
